# BURDEN AND BENEFITS OF INFORMAL CAREGIVING AND OLDER CAREGIVING RECIPIENTS’ SUBJECTIVE WELL-BEING

**DOI:** 10.1093/geroni/igae098.3126

**Published:** 2024-12-31

**Authors:** Weidi Qin, Linh Dang, Briana Mezuk

**Affiliations:** University of Wisconsin Madison, Madison, Wisconsin, United States; University of Michigan, Ann Arbor, Michigan, United States; University of Michigan, Ann Arbor, Michigan, United States

## Abstract

Informal caregiving plays an important role in and has implications for the subjective well-being (SWB) of older caregiving recipients. Using data from the 2021 National Study of Caregiving (N = 991) linked to the National Health and Aging Trend Study (N = 3,321), this study addressed three questions: 1) Is having an informal caregiver associated with better SWB among older adults? 2) Are informal caregivers’ perceptions of caregiving burden and benefits associated with older caregiving recipients’ SWB? 3) Does the relationship between perceived caregiving burden/benefits and caregiving recipients’ SWB differ by gender and relationship types (close family members vs. non-close family)? SWB was measured with a 7-item scale. Caregiving burden was measured with 9 items regarding difficulties in providing caregiving (i.e., emotional difficulty). Caregiving benefit was measured with 4 items indexing beneficial experiences (i.e., closeness to caregiving recipients). Weighted multivariable OLS regressions were used to examine the relationships between caregiving burden, benefit, and SWB. Older adults with a caregiver reported lower levels of SWB compared to those without (B = −0.73, p = 0.001). Expectedly, caregiving burden was associated with lower levels of subjective well-being in caregiving recipients (B = −1.22, p < 0.001). The relationship between caregivers’ perceived benefits and caregiving recipients’ SWB differ by caregiving recipients’ gender (B = 2.01, p = 0.044) and relationship types (B = -2.25, p = 0.032). The findings highlight the importance of considering caregiving recipients’ gender and family relationships in developing interventions to improve informal caregiving experiences.

